# T2* heterogeneity detected by CMR could be related to myocardial iron distribution in Thalassemia patients

**DOI:** 10.1186/1532-429X-11-S1-P134

**Published:** 2009-01-28

**Authors:** Vincenzo Positano, Alessia Pepe, Maria Filomena Santarelli, Anna Ramazzotti, Daniele De Marchi, Antonella Meloni, Eliana Cracolici, Domenico G D'Ascola, Luigi Landini, Massimo Lombardi

**Affiliations:** 1 "G Monasterio" Foundation and Institute of Clinical Physiology, CNR, Pisa, Italy; 2 University of Palermo, Palermo, Italy; 3 A.O. "Bianchi-Melacrino-Morelli", Reggio Calabria, Italy; 4 University of Pisa, Pisa, Italy

**Keywords:** Cardiac Magnetic Resonance, Iron Overload, Surrogate Data, Susceptibility Artefact, Thalassemia Patient

## Introduction

A large percentage of thalassemia major (TM) patients shows a significant heterogeneity of segmental distribution of T2* values in myocardium, measured by multislice multiecho T2* cardiac magnetic resonance (CMR). This finding is in agreement with previous histological studies that have detected heterogeneous iron deposition in hemochromatotic hearts. However, it is not yet clear if that represented true heterogeneous iron density or if it could have been generated by geometric and susceptibility artefacts.

## Purpose

The purpose of this study is to investigate myocardial T2* heterogeneity in TM patients by CMR, and to determine if it is related to inhomogeneous iron overload distribution.

## Methods

230 TM patients consecutively affered to our laboratory were retrospectively studied. Three short-axis views (basal, medium, and apical) of the left ventricle (LV) were obtained by multislice multiecho T2* CMR. T2* segmental distribution was mapped on a 16-segment LV model. The level of heterogeneity of the T2* segmental distribution on each patient was evaluated by the coefficient of variation. Measured heterogeneity was compared with that of a surrogate data set obtained from measurements of subjects without iron overload, to determine whether the inhomogeneous segmental distribution of T2* could be generated only by susceptibility artefacts.

## Results

**Figure 1:**
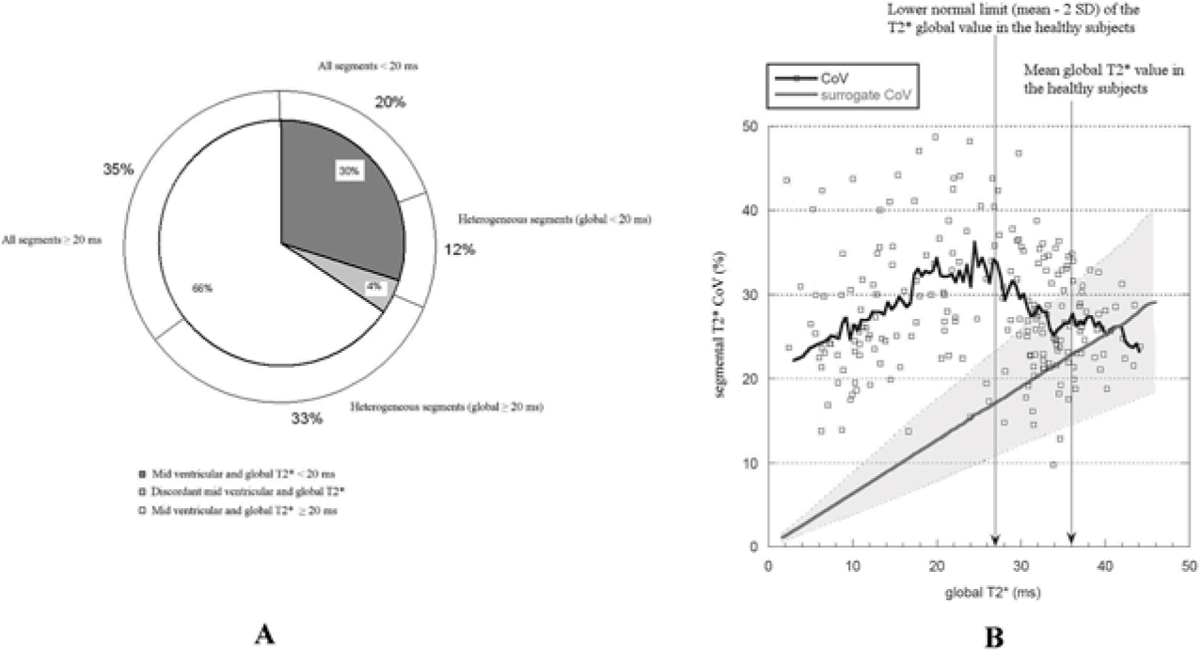
Figure 1

In 45 (20%) TM patients, segmental T2* values were all below the lower limit of normal (20 ms). In 104 (45%) patients, T2* values were heterogeneous with respect to the normal threshold. Of these patients, 74% showed a normal T2* global value. Eighty-one (35%) patients had all normal segments (A). T2* value heterogeneity assessed by the CoV in TM patients and in the surrogate data is shown in Figure 1B. The white squares and the black line represent single patient measurements and the CoV average on all patients, respectively. The grey line represents the mean CoV of the surrogate data with the mean ± 2 SD limits (grey dotted lines). The mean and the normal lower limit of the T2* global value assessed in the healthy subjects are shown as well (vertical black arrows). T2* heterogeneity for patients without iron overload was compatible with the hypothesis that the heterogeneity was generated by susceptibility artefacts. Below the lower limit of normal for global T2*, the heterogeneity abruptly increased and could not be explained by artefactual effects.

## Conclusion

A true heterogeneity in iron overload distribution may be present in TM patients. Heterogeneity seemingly appears in the borderline myocardial iron and stabilizes for moderate to severe iron burden.

